# EIT Observed Hypoxemia Caused by V/Q Mismatch During One‐Lung Ventilation With Indocyanine Green Inhalation: A Report of Two Cases

**DOI:** 10.1002/rcr2.70154

**Published:** 2025-04-24

**Authors:** Xiaoyan Li, Maokun Li, Jian Zhou, Yi Feng, Ke Zhang, Haiyan Xu, Zhenfan Wang, Xue Tian

**Affiliations:** ^1^ Department of Anesthesiology Peking University People's Hospital Beijing People's Republic of China; ^2^ Department of Electronic Engineering Tsinghua University Beijing People's Republic of China; ^3^ Department of Thoracic Surgery Peking University People's Hospital Beijing People's Republic of China; ^4^ Infivision Medical Imaging Technology Co., Ltd Beijing People's Republic of China

**Keywords:** case report, EIT, ICG inhalation, OLV, V/Q

## Abstract

Nebulization under one‐lung ventilation (OLV) can facilitate precise localisation of pulmonary tumours and assist in surgical manipulation. However, hypoxaemia during the nebulization process remains a challenging issue. In this report, we present two cases monitored using Electrical Impedance Tomography (EIT), where individual differences in pulmonary blood flow redistribution contributed to hypoxaemia. This phenomenon was further exacerbated in the lateral decubitus position with the ventilated lung in the upper position. EIT provided real‐time insights into ventilation‐perfusion (V/Q) distribution, offering valuable guidance for managing hypoxaemia during nebulization and surgery under OLV.

## Graphical abstract

In this report, we present two cases monitored using Electrical Impedance Tomography (EIT), where individual differences in pulmonary blood flow redistribution contributed to hypoxemia. This phenomenon was further exacerbated in the lateral decubitus position with the ventilated lung in the upper position. EIT provided real‐time insights into ventilation‐perfusion (V/Q) distribution, offering valuable guidance for managing hypoxemia during nebulisation and surgery under OLV.

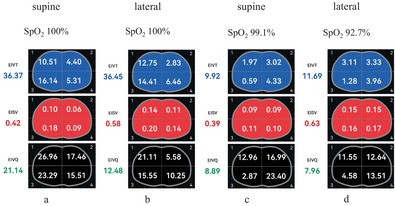

## 1 Introduction

Pulmonary ground‐glass opacity (GGO) and ground‐glass nodule (GGN) present significant challenges in radiological identification due to their minimal visual and tactile differences from surrounding pulmonary tissue. However, accurately determining the tumour margin is critical for achieving precise, minimally invasive resection in patients with invasive pulmonary adenocarcinoma. Indocyanine green (ICG), a fluorescent dye with visualizable properties, has emerged as a valuable tool for intraoperative localization, particularly in thoracic surgery [1]. Compared to traditional methods such as hook‐wire localization, ICG offers a safer and more comfortable alternative [2]. This advantage stems from the selective uptake and retention of ICG by macrophages in healthy pulmonary tissue, creating a clear distinction between tumour and normal tissues.

Inhalation of ICG after anaesthesia induction offers an additional benefit over preoperative intravenous administration. This approach is non‐invasive and safe, with the added advantage of enhancing efficiency and precision in identifying tumour margins. Some studies recommend administering ICG immediately after intubation, before initiating one‐lung ventilation (OLV), to mitigate the effects of lung atelectasis or non‐ventilation on ICG distribution during surgery [3]. Despite these advances, nebulization of ICG solely to the surgical lung may offer further advantages by minimising exposure of the contralateral healthy lung to the dye. Depending on the clinical context, nebulization can be performed in the supine or lateral decubitus position during OLV. However, the effects of these approaches on the patient's ventilation‐perfusion (V/Q) ratio remain poorly understood.

To address this knowledge gap, we observed two patients undergoing ICG nebulization and utilised Electrical Impedance Tomography (EIT) to monitor real‐time pulmonary ventilation and blood flow distribution during the process.

## 2 Case Report

### 2.1 Patients' Information

#### 2.1.1 Case 1

The first patient is a 62‐year‐old female with a height of 152 cm, weight of 39 kg, and a Body Mass Index (BMI) of 16.9. Pulmonary function tests revealed a Forced Expiratory Volume in 1 s (FEV_1_)/Forced Vital Capacity (FVC) ratio of 77.15%. Preoperative Computed Tomography (CT) demonstrated a nodule located in the anterior segment of the right upper lobe, positioned beneath the mediastinal pleura (SE302, IM62). The lesion measured ~14 mm × 10 mm, exhibited poorly defined margins, and caused indentation of the adjacent pleura. It was surrounded by a small area of ground‐glass opacity. Additionally, the patient was noted to have a myocardial bridge in the mid‐portion of the left anterior descending artery, which did not impair her exercise capacity.

#### 2.1.2 Case 2

The second patient is a 70‐year‐old female with a height of 156 cm, weight of 55 kg, and a BMI of 22.6. Pulmonary function tests prior to surgery indicated an FEV_1_/FVC ratio of 61.90%. In July 2023, the patient underwent a left upper lobe lingual segmentectomy. CT imaging revealed multiple ground‐glass opacity nodules in both lungs, with the largest nodule located in the right middle lobe (SE303, IM41, 124), measuring ~15 mm × 9 mm.

### 2.2 Timeline

**FIGURE 1:**
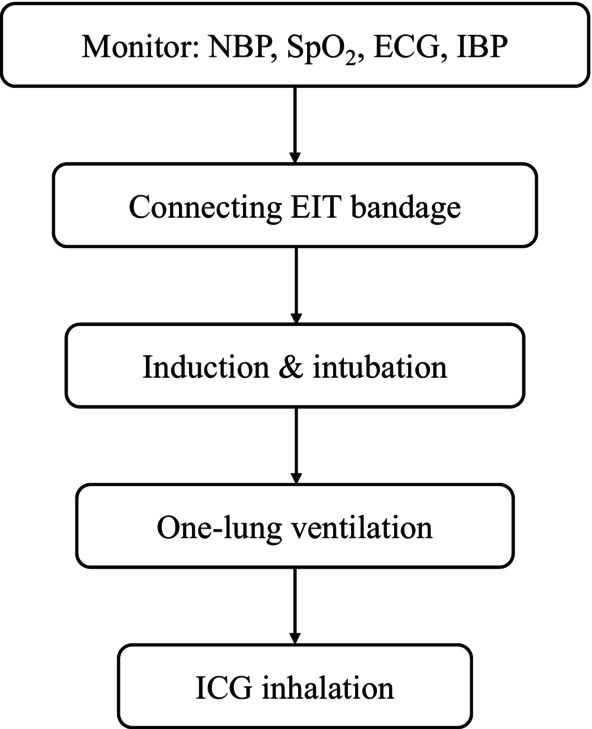
Timeline. ECG, electrocardiography; EIT, electrical impedance tomography; IBP, invasive arterial blood pressure; ICG indocyanine green; NBP, non‐invasive blood pressure; SpO_2_, peripheral oxygen saturation.

Upon the patient's admission to the operating room, standard monitoring is initiated, including non‐invasive blood pressure (NBP), three‐lead electrocardiography (ECG), peripheral oxygen saturation (SpO_2_), and invasive arterial blood pressure (IBP). An Electrical Impedance Tomography (EIT) belt is secured between the third and fourth ribs to enable continuous monitoring. Once the patient is positioned supine, anaesthesia induction is performed, followed by the placement of a double‐lumen endobronchial tube (see Video 1, which demonstrates the proper positioning of the double‐lumen catheter) and the initiation of one‐lung ventilation. After these steps, the patient is transitioned to the lateral decubitus position for indocyanine green (ICG) nebulization inhalation (Figure 1).

**VIDEO 1:** Proper positioning of the double‐lumen catheter. Video content can be viewed at https://onlinelibrary.wiley.com/doi/10.1002/rcr2.70154 Video content can be viewed at https://onlinelibrary.wiley.com/doi/10.1002/rcr2.70154

### 2.3 Clinical Findings

We utilised Electrical Impedance Tomography (EIT) to observe and analyse the clinical findings in both cases.

#### 2.3.1 Case 1

In Case 1, blood flow parameters during supine one‐lung ventilation adapted to the distribution of ventilation, resulting in a favourable matching between pulmonary ventilation and blood flow (see Video 2, which demonstrates the patient's pulmonary blood flow aligning well with ventilation). During lateral decubitus one‐lung ventilation and throughout the process of ICG inhalation, the patient maintained stable oxygenation. In this scenario, nebulization was directed to the lung with the tumour, causing ventilation to shift towards the non‐dependent lung, contrasting with the typical ventilation of the dependent lung during surgery. Despite the effects of gravity necessitating redistribution of pulmonary blood flow, hypoxic pulmonary vasoconstriction effectively optimised V/Q matching. As a result, the patient exhibited no significant hypoxemia during the procedure.

**VIDEO 2:** The patient's pulmonary blood flow aligning well with ventilation. Video content can be viewed at https://onlinelibrary.wiley.com/doi/10.1002/rcr2.70154 Video content can be viewed at https://onlinelibrary.wiley.com/doi/10.1002/rcr2.70154

#### 2.3.2 Case 2

In Case 2, the blood flow parameters during supine one‐lung ventilation failed to adjust in response to changes in ventilation distribution (see Video 3, which shows a lack of pulmonary blood flow adaptation to ventilation alterations). Pulse oxygen saturation progressively declined to 92%. Similarly, during lateral decubitus one‐lung ventilation, oxygen saturation further decreased, necessitating the resumption of two‐lung ventilation to prevent further desaturation. During surgery, one‐lung ventilation was re‐initiated; however, while gravity redirected blood flow to the dependent lung, there remained a poor match between ventilation and perfusion. As a result, the patient's oxygenation status during surgery was suboptimal compared to that of Case 1.

**VIDEO 3:** Lack of pulmonary blood flow adaptation to ventilation alterations. Video content can be viewed at https://onlinelibrary.wiley.com/doi/10.1002/rcr2.70154 Video content can be viewed at https://onlinelibrary.wiley.com/doi/10.1002/rcr2.70154

These findings highlight significant individual variability in pulmonary blood flow redistribution regulated by hypoxic pulmonary vasoconstriction during one‐lung ventilation, which may contribute to hypoxemia in certain patients. EIT proved to be a valuable, non‐invasive, real‐time tool for monitoring these dynamic changes, providing insights into the mechanisms underlying ventilation‐perfusion mismatch and guiding clinical management.

### 2.4 Follow‐Up and Outcomes

#### 2.4.1 Case 1

The patient demonstrated a good match of ventilation and blood flow during one‐lung ventilation nebulization, maintaining stable oxygenation throughout the nebulization and surgical procedures. On the first postoperative day, 90 mL of drainage was collected, followed by 85 mL on the second day. The thoracic drainage tube was removed on the second postoperative day. The surgical wound healed well, and the patient was discharged on the third postoperative day in stable condition.

#### 2.4.2 Case 2

**FIGURE 2:**
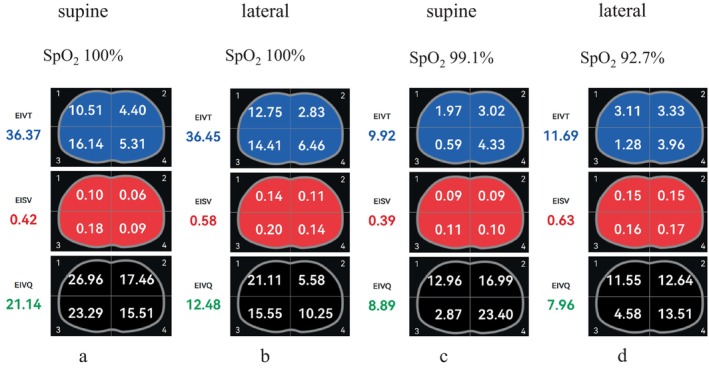
Variations in ventilation and perfusion distribution with body position in the two cases. (a, b): In Case 1, during one‐lung ventilation with nebulization in both the supine and lateral positions, blood flow distribution was well matched with ventilation distribution, maintaining optimal V/Q matching. (c, d): In Case 2, during one‐lung ventilation with nebulization in both the supine and lateral positions, blood flow distribution did not adapt to ventilation distribution, resulting in poor V/Q matching. EIVT: The average tidal volume per breath over a period of 1 min. EISV: The average pulmonary blood flow per heartbeat over a period of 1 min. EIVQ: The ratio of the integral sum of tidal volume over 1 min to the integral sum of pulmonary blood flow over 1 min, calculated using the following formula: EIVQ = (EIVT × RR)/(EISV × HR), where RR = Respiratory Rate (number of breaths per minute) and HR = Heart Rate (number of heart beats per minute).

The patient experienced poor ventilation–perfusion matching during one‐lung ventilation nebulization, which persisted throughout the nebulization and subsequent surgical procedures (Figure 2). Oxygenation levels remained suboptimal compared to Case 1. On the first postoperative day, 175 mL of drainage was collected, followed by 75 mL on the second day. Bedside chest radiography revealed scattered exudative lesions in both lungs and subcutaneous emphysema of the right chest wall. Intermittent drainage was required until the eighth postoperative day, at which point the thoracic drainage tube was removed. The patient was discharged on the ninth postoperative day after a stable recovery.

## 3 Discussion

Transitioning from bilateral lung ventilation to one‐lung ventilation often results in an increased shunt fraction and an imbalance in the ventilation‐perfusion (V/Q) ratio, leading to impaired oxygenation and potential hypoxemia. Hypoxic Pulmonary Vasoconstriction (HPV) is a critical homeostatic mechanism of the pulmonary vasculature, wherein pulmonary arteries constrict in response to alveolar hypoxia. This redirection of blood flow towards better‐ventilated lung segments optimises V/Q matching and systemic oxygenation [4].

Some studies suggest that during lateral decubitus one‐lung ventilation, patients experience improved V/Q matching and oxygenation due to gravity‐driven perfusion to the dependent lung. However, this effect has been debated, as HPV is not considered the primary factor in these findings [5]. Furthermore, gold‐standard methods for evaluating V/Q matching—such as radiologic imaging or invasive techniques—are often impractical in intraoperative settings due to their invasive nature, radiation exposure, and the need for specialised environments and equipment.

Electrical Impedance Tomography (EIT) offers a non‐invasive, radiation‐free, real‐time method to assess V/Q matching intraoperatively. In our observations, Case 2 demonstrated significantly poorer V/Q matching during supine one‐lung ventilation compared to Case 1, as evidenced by EIT (Figure 2). This mismatch was accompanied by worse oxygenation. During lateral decubitus one‐lung ventilation with nebulization, V/Q matching in Case 2 remained poor, leading to significant hypoxemia that necessitated the temporary resumption of bilateral lung ventilation for safety.

Our findings suggest that EIT can provide valuable predictive insights. For instance, if poor V/Q matching is observed during supine one‐lung ventilation, it is likely to persist or worsen during lateral decubitus one‐lung ventilation, thereby increasing the risk of hypoxemia. This highlights EIT's potential as a critical intraoperative tool for monitoring and guiding clinical interventions to optimise patient outcomes.

In conclusion, pulmonary blood redistribution plays a vital role in maintaining oxygenation during one‐lung ventilation, but individual variability exists. This variability may become more pronounced during lateral decubitus one‐lung ventilation with nebulization. EIT provides real‐time insights into V/Q distribution, offering a practical, non‐invasive method to identify and address hypoxemia during nebulization and surgery involving one‐lung ventilation.

This observational study has certain limitations: the placement of the EIT belt within the surgical field necessitated the discontinuation of EIT monitoring prior to surgical disinfection and draping. As a result, any changes in V/Q distribution that might occur during the surgical phase remain unknown. Nevertheless, EIT monitoring during the nebulization phase provided valuable alerts regarding potential hypoxemia risks.

## Author Contributions

**Xiaoyan Li:** data curation, formal analysis, writing – original draft. **Xue Tian:** data curation, formal analysis, methodology, supervision, writing – review and editing. **Yi Feng:** methodology, supervision. **Jian Zhou:** methodology, supervision. **Zhenfan Wang:** data curation. **Maokun Li:** formal analysis, software. **Ke Zhang:** formal analysis, software. **Haiyan Xu:** formal analysis.

## Ethics Statement

The authors attest that the originals of the signed forms are held by the treating institution.

## Consent

The authors attest that the patients described in the manuscript have completed and signed the consent form provided by Respirology Case Reports.

## Conflicts of Interest

The authors declare no conflicts of interest.

## Data Availability

Data sharing not applicable to this article as no datasets were generated or analysed during the current study.
